# Interpretable classification for multivariate gait analysis of cerebral palsy

**DOI:** 10.1186/s12938-023-01168-x

**Published:** 2023-11-22

**Authors:** Changwon Yoon, Yongho Jeon, Hosik Choi, Soon-Sun Kwon, Jeongyoun Ahn

**Affiliations:** 1 Department of Industrial and Systems Engineering, KAIST, Dajeon, South Korea; 2 Department of Applied Statistics/Statistics and Data Science, Yonsei University, Seoul, South Korea; 3 Department of Artificial Intelligence, University of Seoul, Seoul, South Korea; 4 Department of Mathematics/Artificial Intelligence, Ajou University, Suwon, South Korea

**Keywords:** Cerebral palsy, Functional sparse classification, GMFCS, Multivariate functional data, Sparse functional linear discriminant analysis

## Abstract

**Background:**

The Gross Motor Function Classification System (GMFCS) is a widely used tool for assessing the mobility of people with Cerebral Palsy (CP). It classifies patients into different levels based on their gross motor function and its level is typically determined through visual evaluation by a trained expert. Although gait analysis is commonly used in CP research, the functional aspects of gait patterns has yet to be fully exploited. By utilizing the gait patterns to predict GMFCS, we can gain a more comprehensive understanding of how CP affects mobility and develop more effective interventions for CP patients.

**Result:**

In this study, we propose a multivariate functional classification method to examine the relationship between kinematic gait measures and GMFCS levels in both normal individuals and CP patients with varying GMFCS levels. A sparse linear functional discrimination framework is utilized to achieve an interpretable prediction model. The method is generalized to handle multivariate functional data and multi-class classification. Our method offers competitive or improved prediction accuracy compared to state-of-the-art functional classification approaches and provides interpretable discriminant functions that can characterize the kinesiological progression of gait corresponding to higher GMFCS levels.

**Conclusion:**

We generalize the sparse functional linear discrimination framework to achieve interpretable classification of GMFCS levels using kinematic gait measures. The findings of this research will aid clinicians in diagnosing CP and assigning appropriate GMFCS levels in a more consistent, systematic, and scientifically supported manner.

**Supplementary Information:**

The online version contains supplementary material available at 10.1186/s12938-023-01168-x.

## Introduction

Cerebral Palsy (CP) is a group of non-progressive, permanent disorders that cause movement and posture limitations due to disturbances occurring in the fetal or infant brain. Pathological stimuli from the brain result in progressive motor dysfunction and gait disturbances [1, 2]. Gait analysis is a key methodology for studying CP [3], as it is believed that CP patients’ gait deviations are linked to clinical impairments, such as muscle spasticity of lower extremities [4, 5]. The Gross Motor Function Classification System (GMFCS) categorizes CP severity in five levels (1 to 5), with lower levels indicating milder forms and higher levels indicating greater severity [6].

Gait analysis offers objective, three-dimensional (3D) quantitative evaluation of CP patients’ gait pathology. Observing specific deviations in a patient enables more accurate diagnoses and treatment options, such as surgical intervention, physical therapy, and medications. From 3D gait analysis, three kinematic variables are commonly derived and considered most relevant to CP gait pathology: knee flexion/extension angle, hip flexion/extension angle, and ankle dorsiflexion/plantar flexion angle. Note that a flexion angle is defined in the positive direction whereas extension is defined in the negative.

**Fig. 1:**
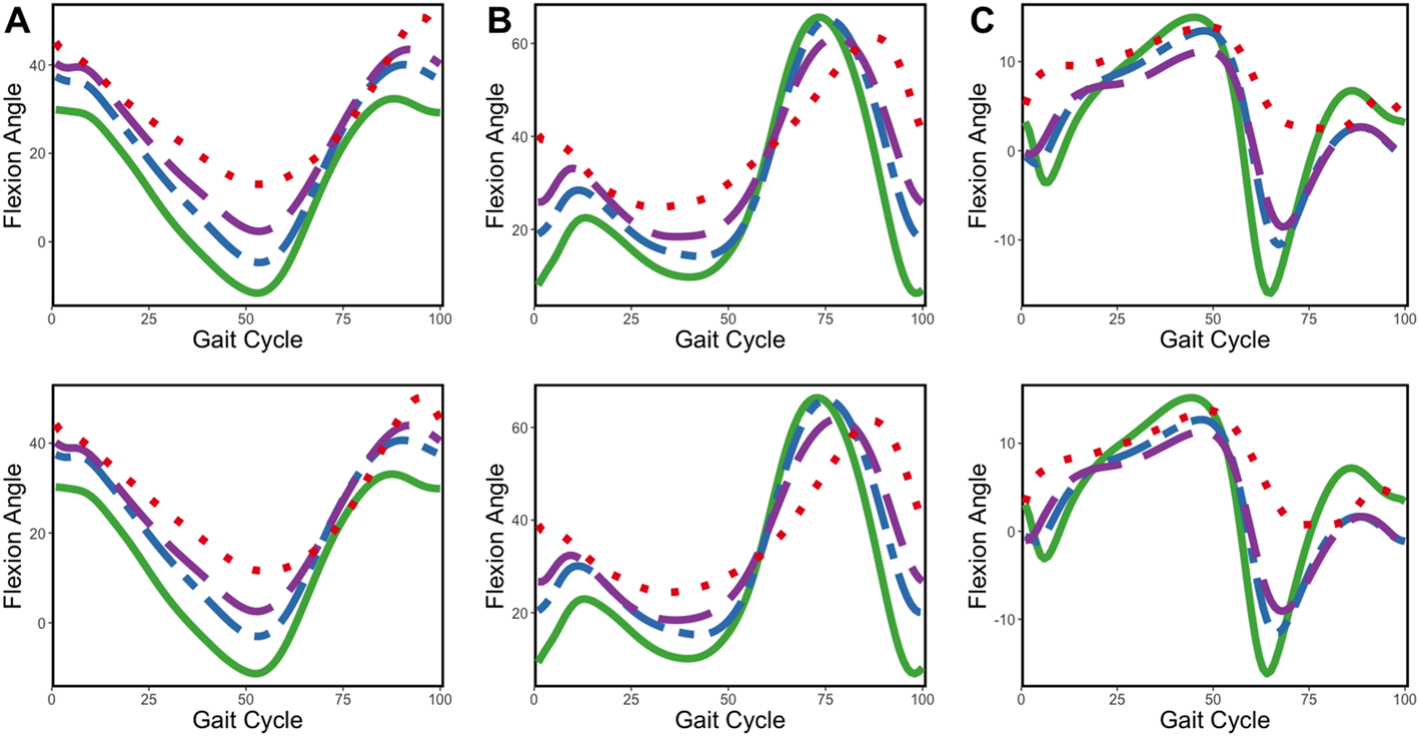
Mean gait patterns of hip, knee, and ankle measures from left (upper) and right (lower), grouped by GMFCS levels. $\textbf{A}$ Hip. $\textbf{B}$ Knee. $\textbf{C}$ Ankle. Level 0 in solid green, level 1 in dot–dashed blue, level 2 in dashed purple, and level 3 in dotted red

As the human gait is inherently continuous, it is natural to present the variables obtained from the gait analysis as curves (functions). Figure 1 shows gait patterns of the three kinematic variables (hip, knee, and ankle flexion angles) in the data used in this study (described in the “Results” section). Each panel displays mean gait patterns for each GMFCS group over a full gait cycle. Level 0 represents the normal group without CP, and the figure displays mean gait patterns from level 0 to 3 since there is only one observation with level 4 (excluded from analysis) and no observation with level 5. A gait cycle is defined as the time from initial contact to the next ipsilateral initial contact and is divided into stance and swing phases: 60% stance and 40% swing [7]. The stance phase is when a person bears weight on a single leg and the swing phase is when he/she advances his/her limb off the floor. The stance phase is further broken down into initial contact, loading response, mid stance, terminal stance, and pre-swing, while the swing phase is broken down into initial swing, mid swing, and terminal swing [8]. From Fig. 1, patients with higher GMFCS levels tend to have reduced changes in hip/knee/ankle flexion angles during both the stance and swing phases. In the stance phase (0–60%), it can be seen that the angle of hip extension tends to decrease, and the angle of the hip/knee joints tends to increase. In other words, in the stance phase, CP patients have a significantly reduced amount of change in maximum hip joint extension and the maximum values of the hip/knee joints are higher than those of normal people. Additionally, it can be seen that CP patients with higher GMFCS levels have a higher maximum hip flexion angle in the gait cycle from the stance to swing phases (50–70%). For CP patients with higher GMFCS levels, the change point in the knee flexion angle appears later during the swing phase, indicating that the movement is unstable. These observed differences between GMFCS levels turn out to be statistically significant. All three kinetic variables yield p values $\le .001$ in the functional ANOVA test [9].

Since each subject’s gait data consists of three functions, the data shown in Fig. 1 is considered multivariate functional data. Despite this, most previous gait analysis studies have not fully utilized its continuous nature. Some studies used a single numerical summary of the gait, such as the Gait Deviation Index (GDI), which measures the deviation of knee, hip, and ankle gait patterns from a normal gait [10]. However, reducing the information in infinite-dimensional functional data to a single numerical value leads to significant information loss. Some studies extracted several numerical gait parameters [11], but they still did not utilize all available information in the gait patterns.

In this study, we propose to analyze gait patterns as multivariate functional data and develop a novel sparse classification model to predict GMFCS levels for CP patients. There have been numerous efforts to associate gait patterns with CP clinical information. Wong et al. [12] suggested a smooth least squares estimator of a gait pattern to identify patient groups with unique clinical characteristics. Zhang and Ma [11] manually extracted seven features from gait patterns and applied several supervised machine learning methods to predict CP subtypes based on clinical observations. Kamruzzaman and Begg [13] utilized stride length and cadence to train Support Vector Machine (SVM) for classifying spastic diplegia CP patients and normal individuals. The work was further developed by Zhang et al. [14] who employed a Bayesian approach to estimate predictive probabilities and hard predictions. Carriero et al. [15] visualized gait parameter subspaces via principal component analysis and found that the normal group formed a distinct cluster, with significant overlap among CP patients. All these studies share limitations in using extracted features and using methods that are designed for Euclidean data.

Recently, there have been new developments in automated gait abnormality detection and classification models. Bajpai et al. [16] utilized neural networks to assess the gait abnormality index developed by Bajpai and Joshi [17] to classify abnormal gait patterns into nine different types. Nguyen and Meunier [18] suggested the gait abnormality index that can be estimated using the adversarial auto-encoder based on sequences of 3D point cloud representations of human gait. Gao et al. [19] used an LSTM–CNN model to classify abnormal gait patterns with gait data collected from wearable sensors. These approaches partially incorporate the continuity of gait patterns and thus show good performance in detecting abnormalities. However, they generally lack interpretability and only focus on classifying normal versus abnormal gait patterns, not considering the gradual severity of the disease.

Building upon the limitations observed in previous studies, our work is motivated by several key objectives:

- **Exploiting the continuous nature of gait data:** Unlike traditional approaches, we treat gait data as multivariate functional data. This enables us to fully capitalize on its continuous nature, as well as incorporate the correlation between different kinetic measurements.
- **Providing clinically relevant interpretations:** A core aim of our work is to produce classification results that are not just accurate but also interpretable in a clinical setting. We believe this adds a valuable layer of applicability to our findings.
- **Detailed gait analysis among varying CP severity levels:** Our work addresses the need for an advanced level of granularity in classifying CP patients according to their GMFCS levels. This is especially crucial for tailoring treatment plans and providing more personalized care.

Functional classification is a supervised learning approach that predicts discrete labels using functions as inputs. Functional Logistic Regression (FLR) [20] and Functional Support Vector Machine (FSVM) [21] are among common techniques. Given a binary response variable $Y \in \{0,1\}$ and functional covariate *X*(*t*), FLR fits a logistic regression model in form of

$$\begin{aligned} \text {log} \left (\frac{\pi }{1-\pi }\right ) = \alpha + \int _{\mathcal {I}} \beta (t)X(t)dt, \end{aligned}$$

where $\pi = E(Y|X(t))$ and $\mathcal {I}$ is a domain of *X*(*t*). Here, $\beta (t)$ is a discriminant function and $\alpha$ is an intercept term. On the other hand, FSVM utilizes standard vectorial SVM after projection of functional covariate to Euclidean $\mathbb {R}^d$ space. The goal of this study is to not only build a classification rule but also to interpret the rule with regards to phases in a gait cycle for a deeper understanding of CP progression. This domain-specific interpretation will aid clinicians in identifying specific areas to focus on during patient examination. We note that existing functional classification methods such as FLR and FSVM cannot identify segments or regions in the function domain that are relevant to the classification task.

Park et al.’s “Sparse Functional Linear Discriminant Analysis” (SFLDA) [22] is a binary classification method for univariate functional data that results in a “sparse” discriminant function, meaning the estimated function is zero where there is no significant difference between groups. This method cannot be applied to our CP gait analysis as the data consists of multi-variable functional data with three functions (flexion/extension angle change of hip, knee, and ankle in the gait cycle) and the GMFCS levels (0, 1, 2, 3) in the data make the problem multi-class. Extending a binary, univariate classification method to a multi-class or multivariate one is not an easy task, even with finite-dimensional Euclidean vectorial data. In this study, we extend the SFLDA framework to multivariate and multi-class classification in the “Methods” section. Our approach is applied to CP gait data, along with comparison to FLR and FSVM and our results are interpreted in the clinical context.

The main contributions of our study are listed as follows.

- **Innovation in functional classification:** We propose an effective, novel functional classification method that is capable of accommodating both multivariate and multi-class functional data.
- **Enhanced interpretability:** Our model is able to identify critical signal regions within the functional domain, which greatly increases the real-world applicability of our work.
- **Insights into kinesiological progression:** By applying our model to gait data gathered from CP patients across various GMFCS levels, we have unveiled distinct kinesiological trends that correlate with increasing GMFCS severity. This adds a new dimension to our understanding of Cerebral Palsy, potentially informing more personalized and effective treatment strategies.

## Results

### Gait data

A 3D gait data set of 833 subjects was collected at Seoul National University Bundang Hospital in South Korea from January 2018 to December 2021. Every procedure involving human participants in this study was conducted in accordance with relevant guidelines and ethical standards. This study was approved by the Institutional Review Board of Seoul National University Bundang Hospital in South Korea (IRB number B-2201-735-101). As the data were obtained retrospectively, Institutional Review Board of Seoul National University Bundang Hospital waived the informed consent. A 3D gait analysis was performed with a Vicon 370 system (Oxford Metrix, Oxford, UK) equipped with 10 cameras and two force plates. Markers were placed as per the Helen Hayes marker [23, 24], which was set by three professional operators who respectively had 22, 5, and 3 years of experience in motion analysis including marker placement. This guaranteed consistent anatomical landmark identification and marker positioning. The three kinematic gait (functional) variables respectively measuring flexion/extension angles at the hip, knee, and ankle at either the left or right side are chosen to be used for our analysis. Each patient walked barefoot on a 10-m walkway more than three times, out of which three trials that represented a patient’s typical gait data were selected. Eventually, the data from the selected three trials were averaged to obtain the gait patterns of each variable. Among the 833 subjects, 500 are normal people without musculoskeletal disorders (GMFCS level 0) at 13 to 76 years of age, 333 are CP patients at 5 to 65 years of age, 133 with GMFCS level 1, 156 with level 2, 43 with level 3, and 1 with level 4. The patient with level 4 is removed from the analysis.

We consider a total of five classification tasks in this study: Three binary classification tasks that compare adjacent levels (0 vs. 1, 1 vs. 2, and 2 vs. 3), a binary task that classifies CP patients from the normal (0 vs. 1,2,3), and a multi-class task that separates the three pathological GMFCS levels 1, 2, and 3, from one another. As for the classification method, we implemented three univariate functional classification methods (SFLDA, FLR, and FSVM) for each of the three measurement variables as well as the proposed multivariate SFLDA (MV SFLDA). Here we present and discuss the results with measurements from the right side only. The results from the left side are almost identical and they are given in the Additional file 1.

### Classification results

**Table 1:** Classification accuracy with standard errors

Variable	Method	0 vs. 1	1 vs. 2	2 vs. 3	0 vs. {1,2,3}	1 vs. 2 vs. 3
R.Hip	SFLDA	0.869 (0.019)	0.673 (0.053)	0.853 (0.038)	0.855 (0.021)	0.637 (0.037)
	FLR	0.897 (0.020)	0.678 (0.052)	0.839 (0.036)	0.910 (0.018)	0.557 (0.038)
	FSVM	0.857 (0.016)	0.593 (0.050)	0.797 (0.013)	0.835 (0.016)	0.534 (0.044)
R.Knee	SFLDA	0.910 (0.018)	0.682 (0.056)	0.870 (0.029)	0.871 (0.021)	0.633 (0.042)
	FLR	0.927 (0.013)	0.685 (0.045)	0.862 (0.042)	0.928 (0.013)	0.563 (0.051)
	FSVM	0.909 (0.015)	0.693 (0.048)	0.877 (0.028)	0.914 (0.017)	0.654 (0.048)
R.Ankle	SFLDA	0.917 (0.015)	0.629 (0.040)	0.844 (0.033)	0.912 (0.016)	0.581 (0.042)
	FLR	0.924 (0.017)	0.627 (0.051)	0.842 (0.043)	0.933 (0.013)	0.505 (0.052)
	FSVM	0.910 (0.022)	0.532 (0.027)	0.793 (0.001)	0.891 (0.022)	0.462 (0.023)
MV SFLDA		0.930 (0.016)	0.672 (0.053)	0.859 (0.033)	0.921 (0.016)	0.619 (0.042)

**Table 2:** False negative rates with standard errors

Variable	Method	0 vs. 1	1 vs. 2	2 vs. 3	0 vs. {1,2,3}	1 vs. 2 vs. 3
R.Hip	SFLDA	0.500 (0.070)	0.308 (0.060)	0.533 (0.132)	0.298 (0.048)	0.397 (0.071)
	FLR	0.343 (0.081)	0.282 (0.058)	0.564 (0.151)	0.147 (0.034)	0.366 (0.077)
	FSVM	0.612 (0.058)	0.370 (0.085)	0.956 (0.087)	0.311 (0.042)	0.647 (0.070)
R.Knee	SFLDA	0.401 (0.089)	0.314 (0.059)	0.511 (0.146)	0.319 (0.052)	0.418 (0.089)
	FLR	0.264 (0.064)	0.304 (0.058)	0.492 (0.139)	0.120 (0.035)	0.363 (0.075)
	FSVM	0.381 (0.062)	0.312 (0.053)	0.525 (0.124)	0.170 (0.045)	0.451 (0.082)
R.Ankle	SFLDA	0.331 (0.078)	0.318 (0.054)	0.603 (0.134)	0.188 (0.037)	0.438 (0.070)
	FLR	0.239 (0.075)	0.328 (0.057)	0.578 (0.158)	0.100 (0.029)	0.418 (0.095)
	FSVM	0.386 (0.098)	0.061 (0.139)	1.000 (0.000)	0.179 (0.044)	0.554 (0.089)
MV SFLDA		0.306 (0.077)	0.303 (0.061)	0.503 (0.138)	0.189 (0.041)	0.417 (0.087)

**Table 3:** False omission rates with standard errors

Variable	Method	0 vs. 1	1 vs. 2	2 vs. 3	0 vs. {1,2,3}	1 vs. 2 vs. 3
R.Hip	SFLDA	0.118 (0.015)	0.358 (0.057)	0.127 (0.028)	0.170 (0.023)	0.233 (0.031)
	FLR	0.085 (0.018)	0.345 (0.058)	0.134 (0.030)	0.093 (0.019)	0.250 (0.031)
	FSVM	0.140 (0.012)	0.439 (0.062)	0.200 (0.014)	0.180 (0.019)	0.310 (0.032)
R.Knee	SFLDA	0.095 (0.019)	0.355 (0.056)	0.120 (0.029)	0.174 (0.024)	0.241 (0.035)
	FLR	0.065 (0.014)	0.347 (0.045)	0.118 (0.031)	0.075 (0.020)	0.253 (0.036)
	FSVM	0.091 (0.013)	0.345 (0.043)	0.122 (0.026)	0.103 (0.024)	0.244 (0.034)
R.Ankle	SFLDA	0.080 (0.017)	0.397 (0.045)	0.140 (0.027)	0.112 (0.020)	0.260 (0.030)
	FLR	0.060 (0.018)	0.403 (0.059)	0.136 (0.031)	0.064 (0.017)	0.276 (0.035)
	FSVM	0.092 (0.021)	0.595 (0.086)	0.207 (0.001)	0.111 (0.025)	0.376 (0.020)
MV SFLDA		0.074 (0.017)	0.357 (0.057)	0.120 (0.029)	0.111 (0.021)	0.243 (0.035)

We report classification performances of the methods in Tables 1, 2, and 3, which respectively report the overall classification accuracy, false negative rates, and false omission rates. False negative rates refer to proportions of patients with higher GMFCS that are classified to a lower level, while false omission rates are how many patients with higher GMFCS are mistakenly present within each estimated label. These metrics may be more relevant than the overall prediction accuracy in the clinical context. For each classification task, metrics were calculated with a random 7:3 split of train and test data, which was repeated 30 times. Within the train data, fivefold cross-validation was implemented to choose the hyperparameters. To avoid potential bias from the unbalanced dataset, each fold was ensured to maintain the same proportion of GMFCS levels as the whole data. Specifically, for SFLDA, the optimal smoothness parameter $\tau$ is searched in the range of [0.0625, 1] and the sparsity parameter $\lambda$ is searched in [0.0156, 0.5]. For FSVM, we used a discretized representation of functions to project functional covariates to a Euclidean vector space, followed by the implementation of a linear SVM using R libraries e1071 [25]. The SVM hyperparameter is tuned in the range of [0.1, 5]. Additionally, we implemented FLR using R package fda.usc [26], treating the number of basis functions as a hyperparameter, with a search space of 3 to 48 with an increment of five. For multi-class classification with FLR, we employed a ‘1 vs. the rest’ strategy.

From the tables, it is clear that discriminating normal vs. CP patients is the easiest problem, while the 1 vs. 2 is the most challenging binary classification task. This implies a significant uncertainty in the GMFCS assignment for milder cases of CP. Among the three univariate methods, FLR seems to be most effective for binary problems while SFLDA is better for the multi-category problem. The proposed MV SFLDA performs comparably to other methods or better in some cases. As for the false negative rates in Table 2 and the false omission rates in Table 3, MV SFLDA and FLR show superior results, while FSVM is the weakest.

### Identification of gait segments relevant to GMFCS classification

In addition to the numerical summaries of the results, a graphical display of the discriminant function ${\hat{\beta }}$ can reveal more detailed information on the classification. Indeed, Davis [27] identified the phases in a gait cycle that characterizes abnormal gaits. Many case studies of CP patients have pointed out the differences in gait patterns between GMFCS levels. For example, Molloy et al. [28] and Malt et al. [29] showed that mean GDI scores are significantly different between GMFCS levels. The latter further specified the kinematic variables such as minimum knee flexion and minimum hip flexion in stance that are different between groups. Also, Robinson et al. [30] revealed that Edinburgh Visual Gait Score (EVGS) which is derived from kinematic components such as knee terminal swing position and hip peak extension swing significantly differs by GMFCS levels. Hence, it would be beneficial to identify the signal regions during the gait cycle that are relevant to the classification between GMFCS levels using the estimated discriminant function.

**Fig. 2:**
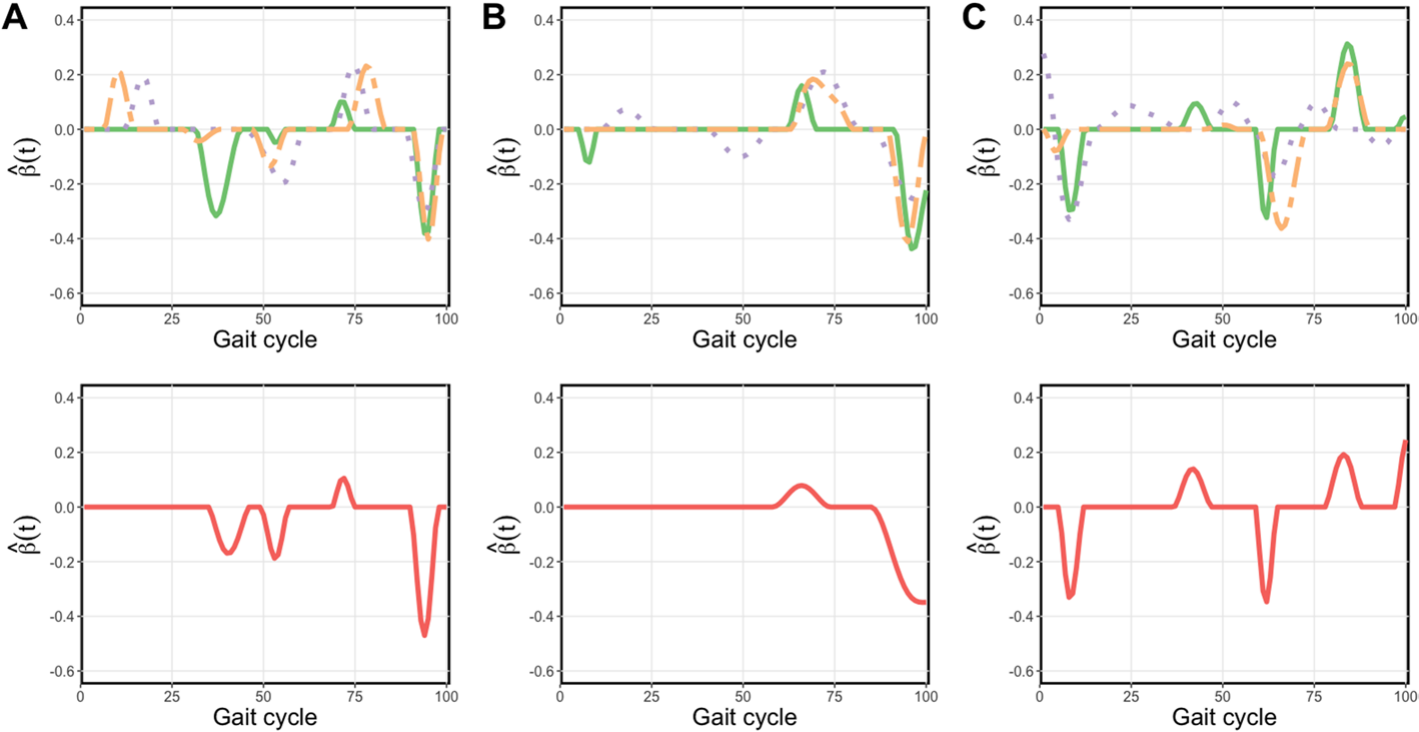
Estimated univariate SFLDA discriminant functions $\hat{\beta }$ from each kinematic variables. $\textbf{A}$ Hip. $\textbf{B}$ Knee. $\textbf{C}$ Ankle. Discriminant functions from binary classification tasks ‘0 vs. 1’, ‘1 vs. 2’, ‘2 vs. 3’ are shown in the first rows, respectively shown in solid green, dotted purple, and dot–dashed orange curves. The $\hat{\beta }$ from the task ‘0 vs. {1,2,3}’ is shown in red in the second row

**Fig. 3:**
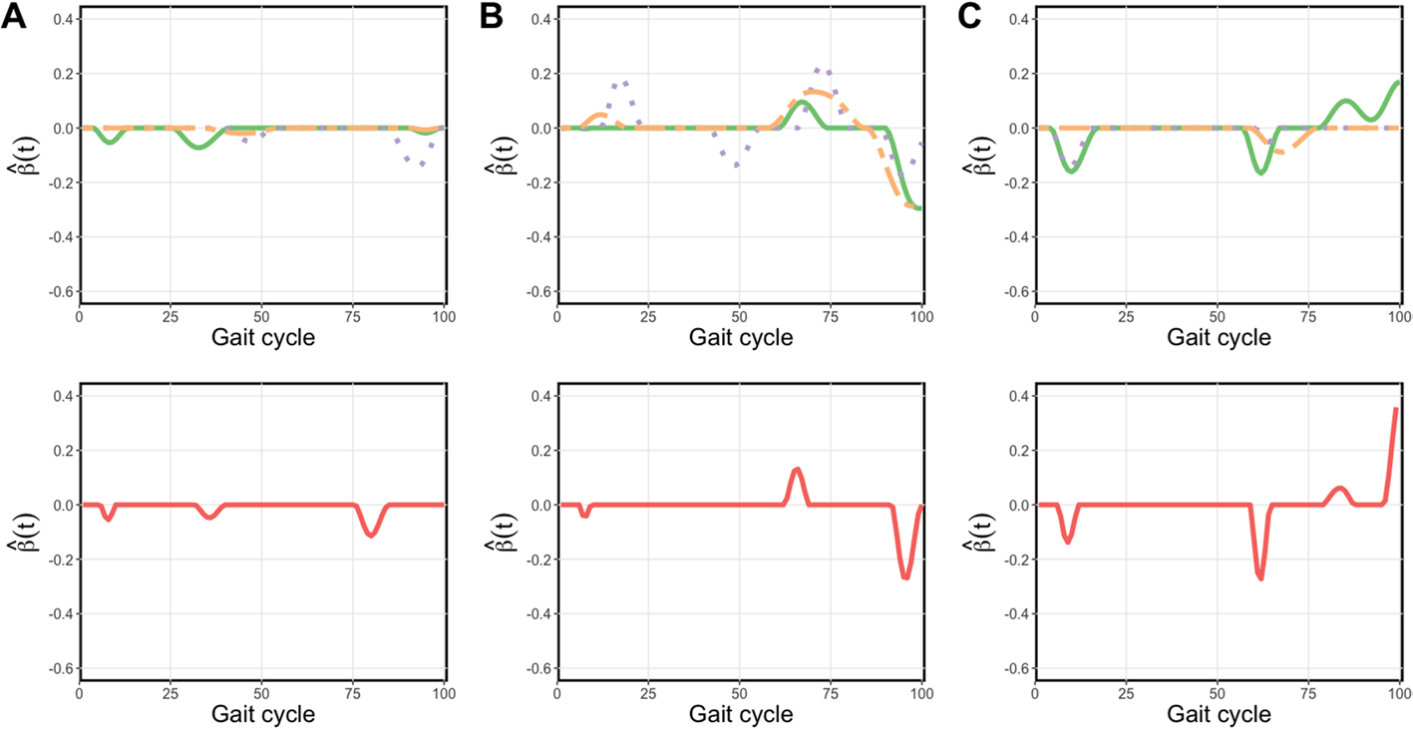
Estimated multivariate discriminant function by MV SFLDA (divided into corresponding kinemtaic variables). **A** Hip. **B** Knee. **C** Ankle. See Fig. 2 for a description

Figure 2 shows the estimated $\hat{\beta }$ function from the univariate SFLDA and Fig. 3 shows the results from MV SFLDA. From these plots, we can identify which phase in the gait cycle is related to the GMFCS level differences. Note that while each curve in Fig. 2 is normalized to have unit $L^2$ norm, i.e., $\Vert \hat{\beta }_j\Vert _2 = 1$, the curves in Fig. 3 are normalized collectively so that $\sum _{j=1}^3\Vert \hat{\beta }_j\Vert _2 = 1$. Due to this distinction, Fig. 3 can reveal the difference in relative contributions among the functional measurements to the classification task. For example, the overall magnitude of $\hat{\beta }_j$ for hip is smaller than knee and ankle, which may indicate that with knee and ankle measurements already utilized for the classification, the additional contribution of hip may not be substantial.

In Fig. 2$\textbf{A}$ and $\textbf{B}$ with $\hat{\beta }$ for hip and knee flexion, we can observe that the discrimination signal is present more in the swing phase than stance phase, and the signal is most prominent in the terminal swing of the gait cycle. This indicates the difficulty for more severe CP patients with limb advancement completed by knee extension and hip anterior flexion. Also in the top panels, we can see that the timing of the difference shows a trend according to the order of comparison. Specifically, the green curve (0 vs. 1) reaches a peak, followed by purple (1 vs. 2) and then orange (2 vs. 3). This trend implies the progress of severity in limb advancement corresponding to GMFCS levels. In Fig. 2$\textbf{C}$ with $\hat{\beta }$ for ankle flexion, it is noticeable that the discrimination signal is large in the loading response section (0–10% of the gait cycle) and the pre-swing section (50–60%).

The multivariate discriminant functions shown in Fig. 3 tend to be more sparse than the curves in Fig. 2. In $\textbf{A}$ with hip, the mid-swing phase (75–87%) seems relevant to the difference between the normal and CP patients, as shown in the red curve in the bottom panel. Normally in the mid-swing phase, the knee and hip flexion get closer and the tibia becomes vertical, which is not the case for CP patients. Especially the terminal-swing region (87–100%) where the purple (1 vs. 2) curve is below zero means that the group with higher severity (GMFCS level 2) is more likely to move only with hip flexion muscles, compared to the group with less severity (GMFCS level 1).

In the middle panels for knee flexion in Fig. 3, the signal region around the terminal swing indicates that a limb advancement completed by knee extension is a good indicator of the severity of CP. Also, the purple curve (1 vs. 2) peak is a bit delayed at around the initial swing phase, which means that the higher severity group (GMFCS level 2) is more likely to suffer from abnormal knee flexion so that their feet are not properly touching the ground. The orange curve (2 vs. 3) peak is over zero means that the group with higher severity (GMFCS level 3) is more likely to move only with knee flexion, compared to the group with less severity (GMFCS level 2) at around the initial phase. This means that it is possible to discriminate by MV SFLDA in the part where the orange curve (2 vs. 3) is almost zero in Fig. 2.

It can also be seen in the right panels for ankle flexion, the green curve (0 vs. 1) and purple curve (1 vs. 2) are below zero in the mid stance phase (15–20%), which indicates that ankle flexion, which is related to whether the foot is properly touching the ground, is more related to the higher severity groups (GMFCS level 1 and GMFCS level 2, respectively). However, the orange curve (2 vs. 3) is only discriminated around the swing phase, which means that the higher severity group (GMFCS level 3) is more likely to suffer from abnormal ankle flexion.

## Conclusions and discussion

Recent advances in technology have made 3D scans readily available in various fields, including medical research. Existing approaches to analyzing 3D gait data mostly rely on several numerical summary measures, to which traditional statistical methods are applied. Even though there have been approaches to train deep neural networks using sequential gait data to detect and classify gait abnormality, they still lack interpretability with respect to the clinical context. In this work, we propose to fully utilize the functional nature of a gait, by developing a multivariate sparse functional classification method. The proposed MV SFLDA method addresses not only covariance structures within each functional variable but also across different variables. The regularization by a smoothness penalty and a sparse penalty is implemented for a smooth discriminant function that selects important regions. By applying the MV SFLDA method to the gait data to solve various GMFCS classification tasks, we could obtain comparable classification performance with other functional classification models. Furthermore, through the estimated sparse discriminant functions, we identified signal regions in the gait cycle that contribute to the classification of GMFCS levels.

It would be worth noting the difference between our approach and rule-based methods which also provide interpretable results. As repeatedly emphasized, our method utilizes infinite dimensionality of the gait data, not relying on a few summarized information. This makes it possible to provide uniquely interpretable classification results, namely, the signal regions within the gait. This aspect differentiates our method’s interpretability from that obtained from rule-based methods. At the same time, it is a purely data-driven approach that does not require external information as rule-based methods do. A possible disadvantage of this machine learning method is that it requires a substantial amount of data to ensure the generalizability of results for future observations.

The common covariance assumption in this work may limit the applicability of the proposed methodology. A simulation study in the setting where the assumption is violated provided evidence for the robustness of our method. Also, the discretization strategy that we adopted for efficient computation could lead to a potential information loss if the underlying functional structure is not smooth. It should also be pointed out that the GMFCS levels (0, 1, 2, 3) are treated as nominal labels in this work, where in fact they are naturally ordered, i.e., they are *ordinal* labels. We partially address their ordinal nature when reporting one-sided classification measures in Tables 2 and 3. Developing an ordinal classification method needs consideration of the ordinality pattern (linear vs. nonlinear) and also the strength of the ordinality [31], which is suggested as a future research topic.

With respect to the interpretation of our results, it is important to clarify the nuances involved. While the GMFCS is generally determined by experts who evaluate the overall gait patterns of CP patients, our study is predicated on analyzing three specific kinematic variables—namely, hip, knee, and ankle flexion angles. Also, it’s worth noting that although the GMFCS is generally regarded as a reliable metric, its assignment process is not entirely objective, particularly when distinguishing between adjacent levels [32, 33]. Therefore, this element of subjectivity should be carefully considered when interpreting the estimated discriminant functions produced by our model.

## Methods

Fisher’s Linear Discriminant Analysis (LDA) is a popular tool for classifying multivariate vector data. Its formulaic versatility has allowed for extensions to non-vector data such as images [34]. It has also been applied to functional data by numerous studies [35–38]. However, many functional LDA methods lack interpretability as they fail to identify the relevant regions in the function domain that contribute to discrimination.

Although SFLDA can effectively generate a smooth discriminant function that is zero in irrelevant areas for classification, its capability is limited to handling binary labeled, univariate functional data. Therefore, we aim to extend this method to accommodate multivariate, multi-class functional gait data. To achieve this, we present a theoretical framework for multivariate functional classification and the optimal classification rule in the first subsection. We use this as the basis for deriving the multivariate extension of SFLDA. Additionally, the second subsection defines a discriminant subspace that is comprised of discriminant functions specifically designed for multi-class classification. For a more detailed explanation of univariate functional linear classification and a theoretical foundation of SFLDA, refer to [22].

### Multivariate SFLDA

Let $X_1, \dots , X_p$ denote functional covariates on domains $\mathcal {I}_1, \dots , \mathcal {I}_p$, respectively, and let *Y* denote the class label, which takes values in $\{1, 2\}$. In the gait data, there are $p = 3$ dimensions, representing flexion/extension angle measurements for hip, knee, and ankle, respectively. The functional covariates all have the same domain, which is one gait cycle for each patient and is denoted as $\mathcal I_j = [0,1]$, $j = 1, \ldots , p$. It is assumed that each covariate curve $X_j$ is square-integrable, i.e., $X_j \in \mathcal L_2(\mathcal I_j)$. The covariance function between the covariates is denoted as $\gamma _{jl}(s,t) = Cov(X_j(s), X_l(t))$ for $j, l = 1, \dots , p$, $s \in \mathcal I_j$, and $t \in \mathcal I_l$. We further assume that the within-class covariance function is the same for both groups, thereby ensuring the appropriateness of the LDA framework.

We aim to find a collection of *p* discriminant functions $\beta = (\beta _1, \dots , \beta _p)$ corresponding to the multivariate functional covariate $X = (X_1, \dots , X_p)$ such that the linear combination

1 $$\begin{aligned} F(X, \beta )=\sum _{j=1}^{p} \int _{\mathcal I_j} X_j(t)\beta _j(t) dt =\sum _{j=1}^{p} \langle X_j, \beta _j \rangle \end{aligned}$$

optimally separates the two groups. Here, $\langle \cdot , \cdot \rangle$ is defined as the standard inner product between two functions in $\mathcal L^2$. Given $\beta$, we use the following classification rule: a given multivariate functional observation *X* is classified as $Y = 1$ if the true classification function $T_0(X)$ is larger than zero, where

$$\begin{aligned} T_0(X) = (F(X,\beta ) - E(F(X, \beta )|Y = 2))^2 - (F(X,\beta ) - E(F(X, \beta )|Y = 1))^2. \end{aligned}$$

The misclassification error of the above classification rule is the probability that the estimated label is different from the true label:

2 $$\begin{aligned} Err = \pi _1 Pr( T_0(X)<0 | Y=1) + \pi _2 Pr( T_0(X) >0 | Y=2), \end{aligned}$$

where $\pi _k = Pr(Y = k)$ is the prior probability of each class with $\pi _1 + \pi _2 = 1$. Suppose the covariates *X* are Gaussian, so that $(\langle X_1, \beta _1 \rangle ,\dots ,\langle X_p, \beta _p \rangle )|Y$ is *p*-variate normal for any $\beta _j \in \mathcal {L}_2(\mathcal {I}_j)$, $j=1,\dots ,p$. Then the error in (2) becomes $Err = 1 - \Phi ( |\delta (\beta )|/(2 \sigma (\beta ))),$ where $\Phi$ is the distribution function of the standard normal, $\delta (\beta ) = E(F(X, \beta )|Y = 2) - E(F(X, \beta )|Y = 1)$ is mean difference and $\sigma (\beta )^2 = Var(F(X,\beta ))$ is variance of the projected covariates. Under the Gaussian setting, the error is minimized when $\beta$ optimizes the following ratio:

3 $$\begin{aligned} \max _\beta \ [\delta (\beta )^2/\sigma (\beta )^2]. \end{aligned}$$

It can be seen that both the numerator and denominator can be expressed dimension-wise: $\delta (\beta )=\sum _{j=1}^{p} \langle \delta _j, \beta _j \rangle$ with $\delta _j(t)=E(X_j(t)|Y=2)-E(X_j(t)|Y=1)$, $j=1,\dots ,p$, and

$$\begin{aligned}\sigma (\beta )^2 = \sum _{j=1}^{p}\sum _{l=1}^{p} Cov(\langle X_j, \beta _j \rangle ,\langle X_l, \beta _l \rangle ) =\sum _{j=1}^{p}\sum _{l=1}^{p} \langle \Gamma _{jl} (\beta _j), \beta _l \rangle , \end{aligned}$$

where $\Gamma _{jl}$ is the covariance operator function defined as

$$\begin{aligned}\Gamma _{jl} (\beta _j) (t) = \int \gamma _{jl}(s,t)\beta _j(s)ds.\end{aligned}$$

The solution to (3) is characterized by the set of equations $\sum _{j=1}^{p} \Gamma _{j l} (\beta _j) = \delta _l, \quad l = 1, \ldots , p,$ which is equivalent to solving

$$\begin{aligned} \min _{\beta } J(\beta ) := \min _{\beta _1,\dots ,\beta _p} ~\frac{1}{2} \sum _{j=1}^{p}\sum _{l=1}^{p} \langle \Gamma _{j l} (\beta _j), \beta _l \rangle - \sum _{j=1}^{p} \langle \delta _{j},\beta _j \rangle . \end{aligned}$$

This formulation allows more flexibility in incorporating regularization penalties [39]. In particular, we aim for smoothness as well as sparsity for the solution. Let $\hat{\beta }$ denote the estimate of $\beta$ based on a finite sample. In order to obtain interpretable $\hat{\beta }$ that can *select* important regions in $\mathcal I_j$, $j = 1, \ldots , p$, we first use the functional $L^1$ norm

$$\begin{aligned} \Vert \beta \Vert _1 := \sum _{j=1}^p \Vert \beta _j\Vert _1 = \sum _{j=1}^p \int _{\mathcal I_j} |\beta _j(t)| dt \end{aligned}$$

that will encourage $\hat{\beta }$ to be exactly zero where there is no meaningful discriminating signal. Another desirable property of $\hat{\beta }$ is smoothness. We use the $L^2$ norm of the derivative of $\beta$:

$$\begin{aligned} \Vert \beta '\Vert ^2_2 := \sum _{j=1}^{p} \Vert \beta _j' \Vert _2^2 = \sum _{j=1}^{p} \int _{\mathcal I_j} (\beta '_j(t))^2 dt. \end{aligned}$$

Thus we propose to solve the following problem with the two regularization terms, after replacing $\Gamma _{jl}$ and $\delta _j$ with the sample estimates:

4 $$\begin{aligned} \min _{\beta _1,\dots ,\beta _p} ~\frac{1}{2} \sum _{j=1}^{p}\sum _{l=1}^{p} \langle \hat{\Gamma }_{j l} (\beta _j), \beta _l \rangle - \sum _{j=1}^{p} \langle \hat{\delta }_{j},\beta _j \rangle + \lambda \sum _{j=1}^{p} \Vert \beta _j \Vert _1 + \tau \sum _{j=1}^{p} \Vert \beta _j' \Vert _2^2, \end{aligned}$$

where $\lambda$ and $\tau$ are tuning parameters that are typically chosen via cross-validation. We denote this method as multivariate SFLDA (MV SFLDA). Let $\hat{\beta }$ denote a solution of (4). The respective roles of the two tuning parameters have been investigated by Park et al. [22]. In short, for a fixed $\lambda$, using a larger $\tau$ will yield a smoother $\hat{\beta }$. On the other hand, for a fixed $\tau$, using a larger $\lambda$ will yield a more spares $\hat{\beta }$, i.e., the set $\biguplus _j\{t_j: \hat{\beta }_j(t_j) = 0\}$ becomes larger.

Note that the proposed method in equation (4) differs from the dimension-wise application of SFLDA. The latter solves the following optimization for each $j=1,\ldots ,p$:

5 $$\begin{aligned} \min _{\beta _j} ~\frac{1}{2} \langle \hat{\Gamma }_{j} (\beta _j), \beta _j \rangle - \langle \hat{\delta }_{j},\beta _j \rangle + \lambda _j \Vert \beta _j \Vert _1 + \tau _j \Vert \beta _j' \Vert _2^2. \end{aligned}$$

We denote a solution to equation (5) by ${\tilde{\beta }}_j$. The key difference between equations (4) and (5) is that equation (4) takes the covariance between different components into account, whereas equation (5) treats each component independently. For our motivating data with CP, it is clear that the gait patterns of the knee, hip, and ankle are correlated, and thus their cross-correlations must be incorporated in the classification process.

The joint estimation approach will also help us determine which component contributes more than others. It’s important to note that in component-wise estimation, the estimated discriminant function $\tilde{\beta }_j$ is typically normalized to have $\Vert \tilde{\beta }_j\Vert _2 = 1$ to ensure stable estimation, as it is a common practice in multivariate analysis to normalize a discriminant vector. As a result, it is difficult to know which functional component is more relevant to the classification task. However, the multivariate functional solution $\hat{\beta }$ may have different magnitudes in each functional variable so that the relative contribution of the variables can be easily deduced.

### Multi-class multivariate sparse functional classification

Our motivating CP gait data has four GMFCS levels. In this section, we discuss how the above multivariate functional classification can be extended to handle multi-class cases where the response variable *Y* takes on *K* values, $K>2$. The goal is to find a subspace spanned by $\beta ^1, \ldots , \beta ^{K-1}$, where each $\beta ^k$ is a collection of *p* discriminant functions. The class assignment is based on the projection of multivariate functions onto the subspace: $(F(X, \beta ^1), \ldots , F(X, \beta ^{K-1}))$, where $F(X, \beta )$ is defined in (1).

The mean difference between the *k*th and *K*th classes, $\delta _j^k = E(X_j(t)|Y = k) - E(X_j(t)|Y = K)$, for $k = 1, \ldots , K-1$, is used to define $\beta ^k$ in the following equations:

$$\begin{aligned} \sum _{j=1}^{p} \Gamma _{j l} (\beta _j^k) = \delta _l^k, \quad l = 1, \ldots , p, \end{aligned}$$

where $\beta _j^k$ corresponds to the *j*th functional component for $\beta ^k$, $k = 1, \ldots , K-1$. For Euclidean data, it has been noted that the optimal subspace can be found by replacing $(\delta _l^1, \ldots , \delta _l^{K-1})$ with any $K-1$ basis functions of span$\{\delta _l^1, \ldots , \delta _l^{K-1}\}$ [39, 40]. Often eigenfunctions are used as a basis, denoted as $\theta _l^1, \ldots , \theta _l^{K-1}$. We propose to find basis functions of $(K-1)$-dimensional discriminant subspace by solving the following for $k = 1, \ldots , K-1$

6 $$\begin{aligned} \begin{aligned} \hat{\beta }^k&= (\hat{\beta }_1, \ldots , \hat{\beta }_p) \\&=\mathop {\textrm{arg min}}\limits _{\beta _1,\dots ,\beta _p} ~\frac{1}{2} \sum _{j=1}^{p}\sum _{l=1}^{p} \langle \hat{\Gamma }_{j l} (\beta _j), \beta _l \rangle - \sum _{j=1}^{p} \langle \hat{\theta }_{j}^k,\beta _j \rangle + \lambda \sum _{j=1}^{p} \Vert \beta _j \Vert _1 + \tau \sum _{j=1}^{p} \Vert \beta _j' \Vert _2^2, \end{aligned} \end{aligned}$$

where the superscript $^k$ is omitted for the sake of simpler notations.

Using eigenfunctions has two advantages in our method. Firstly, solving (6) using $\delta _l^k$ depends on the choice of the base class, which may result in unstable estimates when some classes are not significantly different from each other. However, by using eigenfunctions, this dependency is eliminated. Secondly, the hierarchy of eigenfunctions enables flexible dimension selection in a data-adaptive manner, as shown in [41]. Investigation of the optimization properties and asymptotic classification performance of problems in Eqs. (4) and (6) is suggested as a future work. In the case of $p=1, K=2$, Park et al. [22] established that the misclassification error approaches the Bayes error, $Err_B:= 1-\Phi (\Vert \Gamma ^{-1}\delta \Vert _2/2)$, as the sample size increases.

For the efficient computation of our method, we adopt equidistant grid points for discretization. This widely used approach allows for piecewise linear approximations of each function, with derivative evaluations based on finite-difference approximations. The discretization transforms our problem into a vector-based computation, enabling us to solve it through a lasso-type optimization. Specifically, we employ Fu’s coordinate descent method [42] for this purpose. For a class assignment, we use Fisher’s linear discriminant analysis to the data projected to discriminant subspace.

## Simulation study

**Fig. 4:**
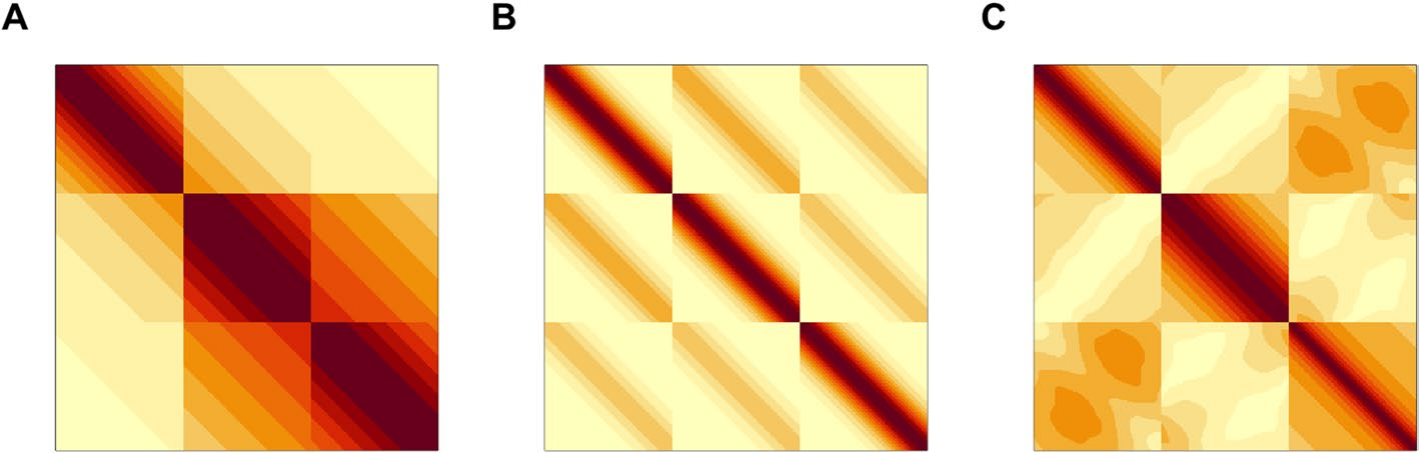
Three covariance structures shown in heatmaps. **A** Type A. **B** Type B. **C** Type C.

**Fig. 5:**
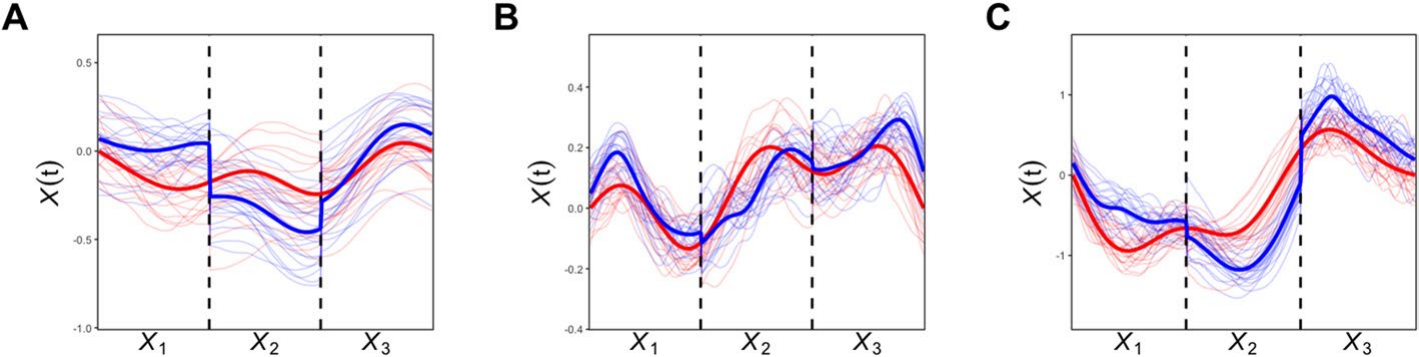
Sample curves from setting 1 with three different covariance structures. **A** Type A. **B** Type B. **C** Type C. Red and blue thick curves are classwise means

We conducted a comparative analysis between the proposed MV SFLDA and some well-known univariate functional classification methods, including FLR and FSVM as well as the original univariate SFLDA, using simulated data. Since a key feature of the multivariate functional data is the dependence among functional variables, we consider three multivariate covariance structures named A, B, and C, for three functional variables $X_1, X_2$, and $X_3$. Heatmaps of the three covariances are in Fig. 4, while the exact formulation can be found in the Additional file 1. The diagonal and off-diagonal parts respectively represent the within and between covariance structures. Under each covariance structure, we consider four settings (settings 1 to 4) that are varied in terms of the sparsity of $\beta$, number of classes *K*, and sample sizes. We assume a common covariance structure between groups for the first four settings. We also consider setting 5 where each group has a different covariance structure. Here, one group has a covariance structure of A while the other has B. These five simulation settings are summarized in Table 4. The rest of the details of the simulation setting can also be found in the Additional file 1. Figure 5 shows sample curves from setting 1.

**Table 4:** Description of simulation settings

Setting	$\beta$	*K*	$n_k$	Common covariance
1	Sparse	2	$(n_1, n_2) = (400,400)$	Yes
2	Sparse	2	$(n_1, n_2) = (200,400)$	Yes
3	Non-sparse	2	$(n_1, n_2) = (400,400)$	Yes
4	Sparse	3	$(n_1, n_2, n_3) = (200,200,200)$	Yes
5	Sparse	2	$(n_1, n_2) = (400,400)$	No

Table 5 reports the average of classification rates (with standard errors) from 100 repetitions evaluated on test data twice the size of the training data. We used fivefold cross-validation to select the hyperparameters based on prediction accuracy within the training data. The consistently superior performance of MV SFLDA suggests a clear benefit of taking the multivariate nature of the data into account. The benefit is most obvious under sparse settings (1 and 2) and under covariance types A and B. Also, from the results for setting 4, we can see that the multi-class extension of SFLDA was also successful, showing comparable performance with other baseline models. It is important to highlight that settings 3 and 5 serve as robustness tests for our proposed model. Specifically, setting 3 challenges the assumption of a sparse discriminant function, while setting 5 tests the model against a common covariance assumption. Despite these intentional violations of foundational assumptions, our MV SFLDA continues to deliver satisfactory performance.

**Fig. 6:**
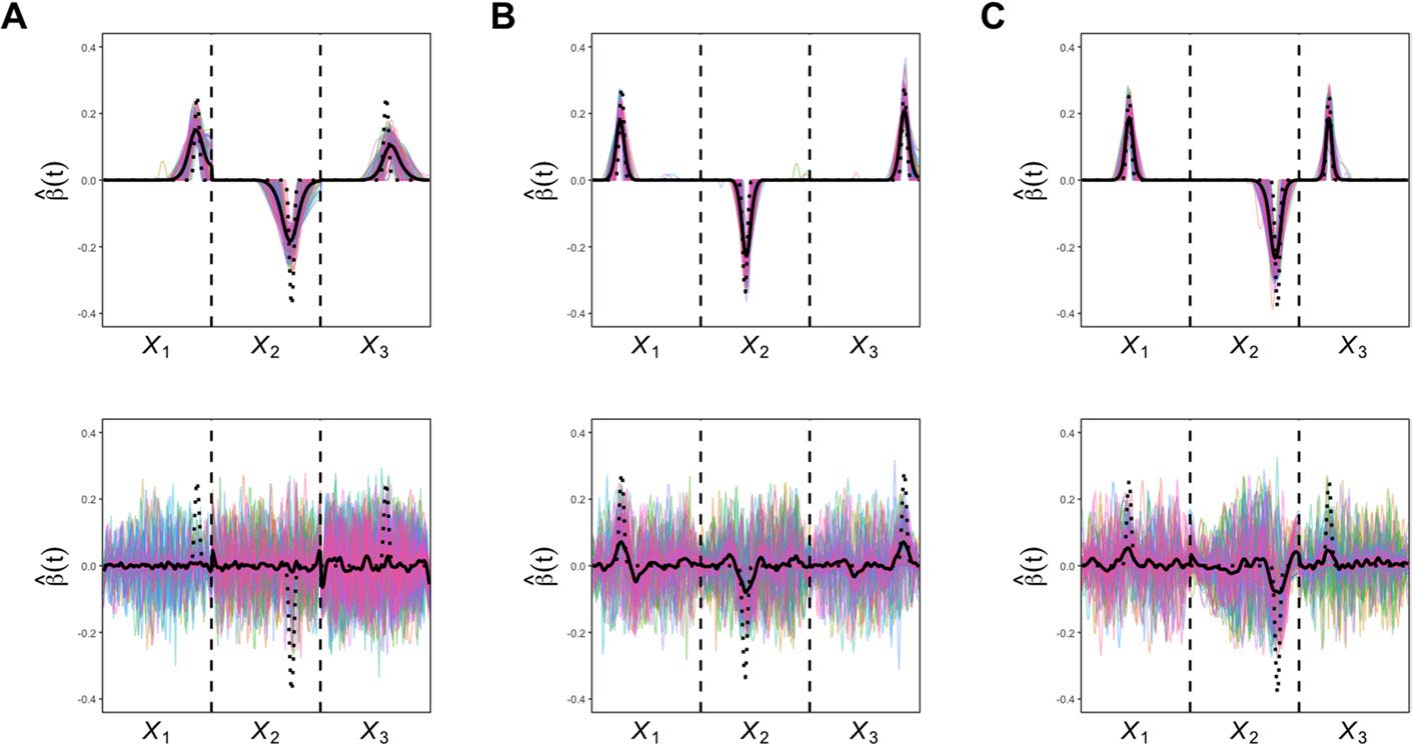
Estimated discriminant functions $\hat{\beta }$ from 100 repetitions for setting 1 with three different covariance structures. **A** Type A. **B** Type B. **C** Type C. Discriminant functions estimated by MV SFLDA are presented in the upper panels and the functions from univariate FLR are presented in the lower panels. Their mean curve is shown in the solid black curve while the true $\beta$ are in the dotted black curve in each panel

A key feature of the proposed MV SFLDA is that the estimated multivariate discriminant function can be sparse, which allows a straightforward interpretation of signal regions. Figure 6 compares the estimated discriminant functions of MV SFLDA and FLR obtained from setting 1. The curves from MV SFLDA in the upper panels successfully identify the true signal regions, while the discriminant functions of FLR fail to do so. Our method allows researchers to pinpoint the specific regions and variables that are crucial for classification tasks. This feature not only facilitates interpretability but also ofurther research. Comparison plots for non-sparse discriminant functions in Setting 3 can be found in the Additional file 1.

**Table 5:** Results of the simulation study. Balanced classification accuracy with standard errors is shown

Variable Method		$X_1$			$X_2$			$X_3$			MV SFLDA
		SFLDA	FLR	FSVM	SFLDA	FLR	FSVM	SFLDA	FLR	FSVM	
Setting 1	A	0.796 (0.010)	0.796 (0.011)	0.794 (0.010)	0.803 (0.011)	0.822 (0.012)	0.791 (0.010)	0.741 (0.012)	0.748 (0.013)	0.701 (0.012)	0.943 (0.005)
	B	0.809 (0.010)	0.812 (0.010)	0.793 (0.010)	0.863 (0.009)	0.863 (0.009)	0.855 (0.009)	0.875 (0.009)	0.876 (0.011)	0.875 (0.008)	0.970 (0.004)
	C	0.902 (0.008)	0.902 (0.007)	0.896 (0.008)	0.935 (0.006)	0.937 (0.007)	0.933 (0.007)	0.885 (0.008)	0.883 (0.009)	0.883 (0.009)	0.972 (0.005)
Setting 2	A	0.776 (0.015)	0.775 (0.016)	0.771 (0.014)	0.783 (0.015)	0.807 (0.013)	0.766 (0.015)	0.712 (0.017)	0.718 (0.016)	0.648 (0.027)	0.939 (0.009)
	B	0.789 (0.015)	0.792 (0.014)	0.770 (0.015)	0.849 (0.012)	0.850 (0.013)	0.839 (0.013)	0.866 (0.012)	0.863 (0.014)	0.865 (0.014)	0.968 (0.006)
	C	0.894 (0.010)	0.892 (0.011)	0.887 (0.011)	0.931 (0.009)	0.932 (0.009)	0.928 (0.009)	0.875 (0.011)	0.872 (0.015)	0.871 (0.013)	0.971 (0.005)
Setting 3	A	0.922 (0.007)	0.932 (0.006)	0.893 (0.008)	0.875 (0.008)	0.872 (0.009)	0.872 (0.008)	0.862 (0.010)	0.861 (0.010)	0.864 (0.009)	0.978 (0.004)
	B	0.797 (0.010)	0.797 (0.009)	0.798 (0.009)	0.880 (0.008)	0.882 (0.008)	0.867 (0.009)	0.910 (0.008)	0.910 (0.008)	0.912 (0.008)	0.948 (0.006)
	C	0.738 (0.012)	0.737 (0.012)	0.726 (0.011)	0.747 (0.012)	0.752 (0.012)	0.737 (0.011)	0.752 (0.012)	0.752 (0.013)	0.753 (0.012)	0.900 (0.008)
Setting 4	A	0.914 (0.009)	0.913 (0.008)	0.911 (0.009)	0.882 (0.011)	0.893 (0.009)	0.855 (0.011)	0.650 (0.016)	0.675 (0.016)	0.619 (0.012)	0.981 (0.005)
	B	0.750 (0.015)	0.745 (0.014)	0.724 (0.014)	0.857 (0.011)	0.856 (0.011)	0.815 (0.013)	0.713 (0.013)	0.705 (0.013)	0.688 (0.013)	0.957 (0.006)
	C	0.825 (0.012)	0.824 (0.011)	0.757 (0.021)	0.941 (0.007)	0.946 (0.007)	0.930 (0.007)	0.890 (0.010)	0.886 (0.010)	0.879 (0.010)	0.985 (0.004)
Setting 5	A & B	0.791 (0.011)	0.794 (0.010)	0.777 (0.010)	0.833 (0.012)	0.850 (0.013)	0.827 (0.012)	0.746 (0.014)	0.757 (0.013)	0.749 (0.013)	0.925 (0.007)

### Supplementary Information

**Additional file 1.** Additional tables and figures.

**Additional file 2.** Simulation codes.

## Data Availability

The clinical data underlying this article are unavailable to the public and non-shareable since Institutional Review Board (IRB) approved the patient data for the analysis purpose only. Any inquiries regarding the clinical data can be directed to the corresponding author, Soon-Sun Kwon (qrio1010@ajou.ac.kr). The R implementation of SFLDA model is available at https://github.com/cwyoon96/Sparse-Functional-Linear-Discriminant-Analysis-SFLDA-. Source codes for the simulation study is available as an additional file of the article.

## Abbreviations

CP: Cerebral Palsy
GMFCS: Gross Motor Function Classification System
3D: Three-dimensional
GDI: Gait Deviation Index
SVM: Support Vector Machine
FLR: Functional Logistic Regression
FSVM: Functional Support Vector Machine
SFLDA: Sparse Functional Linear Discriminant Analysis
LDA: Linear Discriminant Analysis
MV SFLDA: Multivariate SFLDA
EVGS: Edinburgh Visual Gait Score
