# Supplementary material for: Interpretable classification for multivariate gait analysis of cerebral palsy
Source: Biomed Eng Online. 2023 Nov 22;22:109. doi: 10.1186/s12938-023-01168-x (PMC10664661; doi:10.1186/s12938-023-01168-x)
Supplement: Supplementary file 1 — Additional file 1. Additional tables and figures. [file 12938_2023_1168_MOESM1_ESM.pdf]

Supplementary Materials for Interpretable Classification for Multivariate Gait Analysis of Cerebral Palsy

This document includes supplementary figures and tables, along with information for the generation of synthetic data.

1 Classification results with kinematic variables from the left

In this section, we present classification results with kinematic variables from the left-side measurements. Classification accuracy along with false negative rate and false omission rate are shown in Tables S1, S2, and S3, respectively. We also present estimated discriminant functions using SFLDA in Figures S1 S2. They generally show similar results to the analysis using the right-side measurements presented in the paper, due to strong correlations between left- and right-side measurements.

Table S1: Classification accuracies

Variable	Method	0 vs. 1	1 vs. 2	2 vs. 3	0 vs. {1,2,3}	1 vs. 2 vs. 3
	SFLDA	0.867 (0.018)	0.653 (0.051)	0.870 (0.034)	0.835 (0.018)	0.635 (0.037)
L.Hip	FLR	0.885 (0.023)	0.678 (0.041)	0.865 (0.034)	0.894 (0.017)	0.589 (0.044)
	FSVM	0.835 (0.012)	0.644 (0.049)	0.796 (0.012)	0.820 (0.017)	0.580 (0.047)
	SFLDA	0.917 (0.016)	0.696 (0.050)	0.855 (0.042)	0.875 (0.018)	0.629 (0.041)
L.Knee	FLR	0.935 (0.014)	0.685 (0.040)	0.856 (0.043)	0.929 (0.013)	0.566 (0.041)
	FSVM	0.923 (0.014)	0.684 (0.043)	0.857 (0.035)	0.920 (0.016)	0.625 (0.047)
	SFLDA	0.923 (0.017)	0.616 (0.038)	0.863 (0.038)	0.914 (0.013)	0.578 (0.034)
L.Ankle	FLR	0.943 (0.015)	0.616 (0.039)	0.878 (0.038)	0.939 (0.013)	0.542 (0.030)
	FSVM	0.906 (0.017)	0.531 (0.029)	0.793 (0.001)	0.912 (0.016)	0.472 (0.039)
MV SFLDA		0.938 (0.014)	0.692 (0.044)	0.865 (0.044)	0.914 (0.016)	0.625 (0.037)

Table S2: False negative rates

Variable	Method	0 vs. 1	1 vs. 2	2 vs. 3	0 vs. {1,2,3}	1 vs. 2 vs. 3
	SFLDA	0.506 (0.066)	0.320 (0.084)	0.472 (0.128)	0.351 (0.039)	0.388 (0.058)
L.Hip	FLR	0.374 (0.080)	0.309 (0.070)	0.489 (0.125)	0.166 (0.045)	0.319 (0.073)
	FSVM	0.730 (0.047)	0.349 (0.074)	0.972 (0.063)	0.358 (0.036)	0.636 (0.065)
	SFLDA	0.370 (0.064)	0.311 (0.050)	0.489 (0.127)	0.304 (0.044)	0.405 (0.071)
L.Knee	FLR	0.245 (0.063)	0.304 (0.070)	0.450 (0.136)	0.124 (0.025)	0.375 (0.067)
	FSVM	0.317 (0.065)	0.358 (0.064)	0.519 (0.148)	0.170 (0.040)	0.444 (0.082)
	SFLDA	0.295 (0.081)	0.338 (0.073)	0.550 (0.160)	0.187 (0.036)	0.450 (0.061)
L.Ankle	FLR	0.183 (0.059)	0.348 (0.057)	0.456 (0.168)	0.095 (0.029)	0.357 (0.086)
	FSVM	0.362 (0.071)	0.107 (0.146)	1.000 (0.000)	0.170 (0.041)	0.547 (0.074)
MV SFLDA		0.268 (0.068)	0.300 (0.067)	0.447 (0.125)	0.210 (0.038)	0.398 (0.070)

Table S3: False omission rates

Variable	Method	0 vs. 1	1 vs. 2	2 vs. 3	0 vs. {1,2,3}	1 vs. 2 vs. 3
	SFLDA	0.120 (0.014)	0.374 (0.065)	0.113 (0.028)	0.194 (0.018)	0.233 (0.028)
L.Hip	FLR	0.092 (0.018)	0.352 (0.05)	0.117 (0.026)	0.104 (0.024)	0.233 (0.032)
	FSVM	0.162 (0.009)	0.390 (0.056)	0.203 (0.010)	0.201 (0.016)	0.291 (0.031)
	SFLDA	0.088 (0.014)	0.343 (0.049)	0.118 (0.028)	0.168 (0.020)	0.240 (0.031)
L.Knee	FLR	0.061 (0.015)	0.345 (0.047)	0.111 (0.030)	0.078 (0.014)	0.258 (0.035)
	FSVM	0.077 (0.015)	0.364 (0.043)	0.123 (0.030)	0.102 (0.022)	0.259 (0.036)
	SFLDA	0.072 (0.018)	0.412 (0.051)	0.128 (0.033)	0.111 (0.018)	0.267 (0.026)
L.Ankle	FLR	0.046 (0.014)	0.416 (0.044)	0.108 (0.036)	0.061 (0.017)	0.255 (0.033)
	FSVM	0.088 (0.016)	0.546 (0.156)	0.207 (0.001)	0.103 (0.022)	0.374 (0.072)
MV SFLDA		0.065 (0.016)	0.339 (0.054)	0.109 (0.028)	0.122 (0.020)	0.239 (0.034)

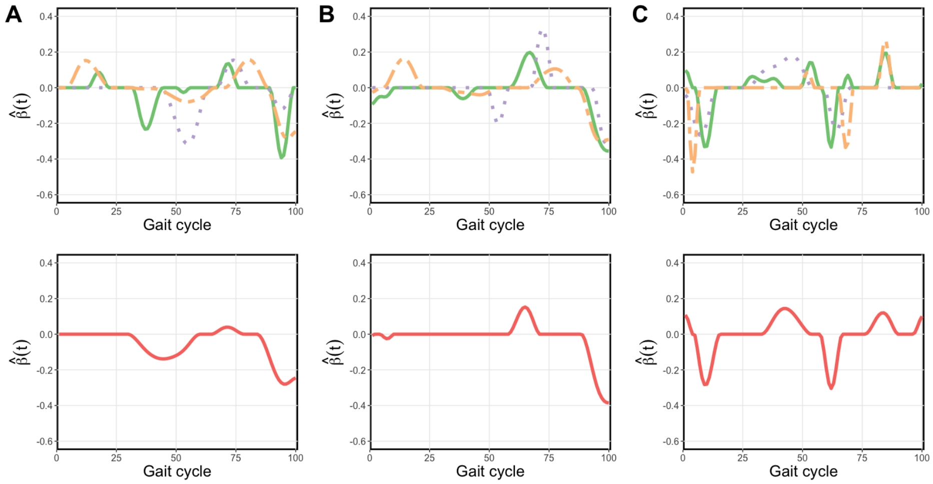

Figure S1: The estimated univariate SFLDA discriminant functions $\hat{\beta}$ from each kinematic variables. **A** Hip. **B** Knee. **C** Ankle. Discriminant functions from binary classification tasks '0 vs. 1', '1 vs. 2', '2 vs. 3' are shown in the first rows, respectively shown in solid green, dotted purple, and dot-dashed orange curves. The $\hat{\beta}$ from the task '0 vs. {1,2,3} is shown in red in the second row.

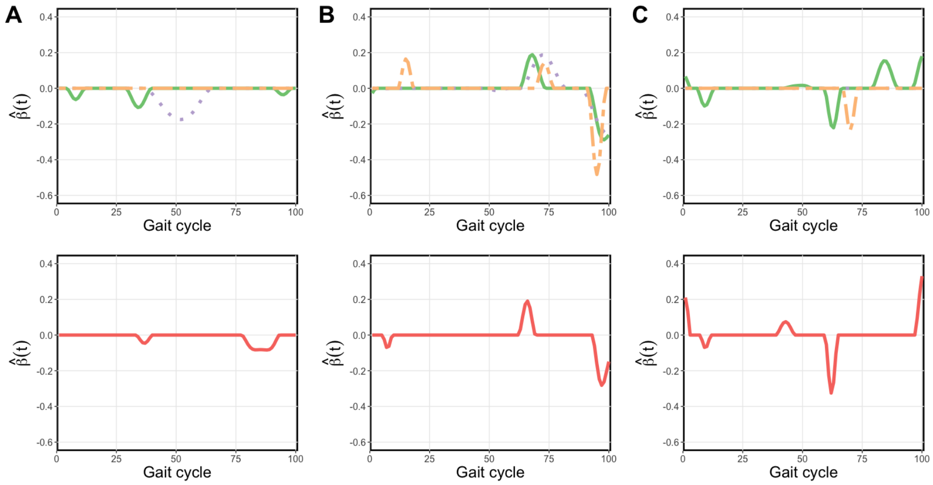

Figure S2: The estimated multivariate discriminant function by MV SFLDA (divided into corresponding kinematic variables). **A** Hip. **B** Knee. **C** Ankle. Descriptions are the same with previous figure.

2 Details of synthetic data generation

In this section, we address the details of synthetic data generation for simulation studies. For simplicity, consider the concatenated version of β_j, denoted as $\boldsymbol{\beta}(t)$, $t \in \biguplus_j \mathcal{I}_j$, which is created by simply linking $\beta_1, \ldots, \beta_p$ without connecting them at the knots, thus allowing discontinuity points at $p-1$ knots. Define $\mathbf{X}(t)$ and $\boldsymbol{\delta}(t)$ in a similar way, and let $\boldsymbol{\Gamma}$ be the covariance operator

$$\boldsymbol{\Gamma}(\boldsymbol{\beta})(t) = \int_{\biguplus_j \mathcal{I}_j} \boldsymbol{\gamma}(s,t)\boldsymbol{\beta}(s)ds,$$

where $\boldsymbol{\gamma}(s,t) = Cov(\mathbf{X}(s), \mathbf{X}(t))$.

2.1 Construction of population covariance structures

In the simulation study, three different trivariate covariance structures (A, B, and C) were used. Let $\boldsymbol{\gamma}_1, \boldsymbol{\gamma}_2$ and $\boldsymbol{\gamma}_3$ denote the covariance function for A, B and C respectively. We constructed $\boldsymbol{\gamma}_1$ and $\boldsymbol{\gamma}_2$ using Matérn covariance functions while construction of $\boldsymbol{\gamma}_3$ was inspired from Chiou et. al. (2014) where we use Gaussian covariance function and rational quadratic covariance function along with Matérn covariance function.

Matérn covariance function is defined as

$$m(s,t) = \sigma^2 \frac{2^{1-\nu}}{G(\nu)} \bigg\{ (2\nu)^{1/2} \frac{|s-t|}{\rho} \bigg\}^{\nu} K_\nu \bigg\{ (2\nu)^{1/2} \frac{|s-t|}{\rho} \bigg\}.$$

Here, G and K_ν denote the gamma function and the modified Bessel function of the second kind respectively, while σ, ρ, ν are parameters controlling the covariance structure. Gaussian covariance function is defined as

$$g(s,t) = \sigma^2 \exp\{-(|s-t|/V)^2\},$$

where σ and V are parameters for the covariance structure. Finally, the rational quadratic covariance function is defined as

$$r(s,t) = \bigg(1 + \frac{|s-t|^2}{2\alpha k^2}\bigg)^{-\alpha},$$

with parameters α and k.

Since the implementation of SFLDA used a discretization strategy, covariance matrices, $\widetilde{\gamma}_1, \widetilde{\gamma}_2, \widetilde{\gamma}_3$, were constructed from discretization of covariance functions. Consider T equispaced grid points $t_i, i = 1, ..., T$, on $[0,1]$. With these grid points, $T \times T$ covariance matrix, $\widetilde{\gamma}$, can be constructed where (i,j) entry equals $\gamma(t_i, t_j)$. Here, γ can be Matérn, Gaussian, or rational quadratic covariance function. A distinctive feature of multivariate covariance structure is that the covariance matrix is in blocks, each block representing the covariance matrix among covariates. We discretize our synthetic functional data from one covariate with 100 equispaced points. Hence, our final trivariate covariance matrices will be 300×300 matrices with 9 blocks.

2.1.1 Construction of $\widetilde{\gamma}_1$

One way to construct a block matrix is to use a combination of block diagonal matrices. First, we construct covariance matrices, $\widetilde{\gamma}_1, ..., \widetilde{\gamma}_5$, using Matérn covariance function with the parameters given in Table S4.

Table S4: Parameters for each blocks.

	T	σ	ρ	ν
$\widetilde{\gamma}_1$	200	1.5	0.3	2
$\widetilde{\gamma}_2$	200	2	0.7	2
$\widetilde{\gamma}_3$	100	1.5	0.5	3
$\widetilde{\gamma}_4$	100	2	0.5	2
$\widetilde{\gamma}_5$	300	3	3	4

Then, compose a 300×300 block diagonal matrix C_1 with $\widetilde{\gamma}_1$ and $\widetilde{\gamma}_3$,

$$C_1 = \begin{bmatrix} \widetilde{\gamma}_1 & 0 \\ 0 & \widetilde{\gamma}_2 \end{bmatrix}.$$

Likewise, compose another block diagonal matrix C_2 with $\widetilde{\gamma}_4$ and $\widetilde{\gamma}_2$,

$$C_2 = \begin{bmatrix} \widetilde{\gamma}_4 & 0 \\ 0 & \widetilde{\gamma}_2 \end{bmatrix}.$$

Finally, $\widetilde{\gamma}_5$ itself compose C_3. We now construct the 300×300 trivariate covariance structure as $\widetilde{\boldsymbol{\gamma}}_1 = 0.25C_1 + 0.25C_2 + 0.5C_3$. Note that $\widetilde{\boldsymbol{\gamma}}_1$ is then scaled to have values between 0 and 1 for stabilization.

2.1.2 Construction of $\widetilde{\boldsymbol{\gamma}}_2$

Another way to construct block-wise multivaraite covariance matrix is through Kronecker product. Let $\widetilde{\gamma}_{\text{base}}$ be a Matérn covariance matrix with parameters, $T = 100, \sigma = 1, \rho = 0.2, \nu = 3$. Also, define 3×3 extension matrix as

$$E = \begin{bmatrix} 1 & 0.4 & 0.3 \\ 0.4 & 1 & 0.3 \\ 0.3 & 0.3 & 1 \end{bmatrix}.$$

Then, 300×300 trivariate covariance structure $\widetilde{\boldsymbol{\gamma}}_2$ is constructed as

$$\widetilde{\boldsymbol{\gamma}}_2 = E \otimes \widetilde{\gamma}_{\text{base}}.$$

Note that $\widetilde{\boldsymbol{\gamma}}_2$ is also scaled to have values between 0 and 1.

2.1.3 Construction of $\widetilde{\boldsymbol{\gamma}}_3$

As noted earlier construction of $\widetilde{\boldsymbol{\gamma}}_3$ is mostly inspired from the simulation settings introduced in Chiou et. al. (2014). Let $\widetilde{\gamma}_{jk}$ indicates (j, k)th block of the matrix $\widetilde{\boldsymbol{\gamma}}_3$. That is,

$$\widetilde{\boldsymbol{\gamma}}_3 = \begin{bmatrix} \widetilde{\gamma}_{11} & \widetilde{\gamma}_{12} & \widetilde{\gamma}_{13} \\ \widetilde{\gamma}_{21} & \widetilde{\gamma}_{22} & \widetilde{\gamma}_{23} \\ \widetilde{\gamma}_{31} & \widetilde{\gamma}_{32} & \widetilde{\gamma}_{33} \end{bmatrix},$$

where $\widetilde{\gamma}_{jk}$ is a 100×100 matrix. Then we construct the block-wise $\widetilde{\boldsymbol{\gamma}}_3$ as follows.

1. Set $\widetilde{\gamma}_{jj}, j = 1, 2, 3$, as Matérn covariance matrix with $T = 100, \sigma = 1, \rho = 0.2, \nu = 3$, Gaussian covariance matrix with $T = 100, \sigma = 1, V = 0.5$ and rational quadratic covariance matrix with $T = 100, k = 0.1, \alpha = 0.5$ respectively. Also, obtain eigenvalues and eigenvectors of each $\widetilde{\gamma}_{jj}$ denoted as λ_r and ν_{jr} where $\widetilde{\gamma}_{jj} = \sum_{r=1}^{100} \lambda_{jr} \nu_{jr} \nu_{jr}^T$.
2. Now, construct the cross-covariance structure as $\widetilde{\gamma}_{jk} = \sum_{r=1}^{100} \hat{\lambda}_r \nu_{jr}^* \nu_{kr}^{*\,T}$, $j \neq k$, where $\hat{\lambda}_r = \sum_{j=1}^{3} \lambda_{jr}/3$, $\nu_{jr}^* = \nu_{jr}/\sqrt{3}$ and $\nu_{kr}^* = \nu_{kr}/\sqrt{3}$.
3. Note that the constructed $\widetilde{\boldsymbol{\gamma}}_3$ is not positive semi-definite, which implies that it can not truly represent the covariance matrix. Hence, we reconstruct $\widetilde{\boldsymbol{\gamma}}_3$ using only the positive eigenvalues and corresponding eigenvectors.

2.2 Simulation settings

Along with the trivariate covariance structure, the true multivariate discriminant function, $\boldsymbol{\beta}(t)$, and multivariate mean function for the first class, $\boldsymbol{\mu}^0(t)$, were also constructed. Then, the mean function for the second class was constructed as $\boldsymbol{\mu}^1(t) = \boldsymbol{\mu}^0(t) + \boldsymbol{\delta}(t)$. Here, $\boldsymbol{\delta}(t)$ is a multivariate mean difference function obtained from $\boldsymbol{\delta}(t) = \int_{\biguplus_j \mathcal{I}_j} \boldsymbol{\gamma}(t, s)\boldsymbol{\beta}(s)ds$. (We used $\boldsymbol{\gamma}_{12}(t, s) = \frac{1}{2}(\boldsymbol{\gamma}_1(t, s) + \boldsymbol{\gamma}_2(t, s))$ to obtain $\boldsymbol{\delta}(t)$ for setting 5.) In multi-class case, we considered three classes which implies that we need two multivariate discriminant functions, denoted as $\boldsymbol{\beta}^k(t), k = 1, 2$. Then, two multivariate mean difference function were obtained by $\boldsymbol{\delta}^k(t) = \int_{\biguplus_j \mathcal{I}_j} \boldsymbol{\gamma}(t, s)\boldsymbol{\beta}^k(s)ds$. Finally, the mean functions for the second and third classes were constructed as $\boldsymbol{\mu}^k(t) = \boldsymbol{\mu}^0(t) + \boldsymbol{\delta}^k(t)$, respectively.

Finally, the samples for each class ($m = 0, 1$, or 2 for multi-class) were generated as $\boldsymbol{x}^m(t) = \boldsymbol{\mu}^m(t) + \int_{\biguplus_j \mathcal{I}_j} \boldsymbol{\gamma}^{1/2}(t, s)\boldsymbol{e}^m(s)ds$ where $\boldsymbol{e}^m(s)$ are random noise generated from Gaussian distribution with mean 0 and standard deviation τ which is a parameter to set.

Similar to the covariance structure, we discretized $\widetilde{\boldsymbol{\beta}}$ and $\widetilde{\boldsymbol{\mu}}$. Discriminant functions, $\beta_j(t)$, were defined for each covariate and then concatenated to form a multivariate discriminant function, $\boldsymbol{\beta}(t)$. For sparse discriminant function, 33 cubic B-spline basis functions, $B_{i,4}, i = 1, ..., 33$, with 31 knots equispaced in [0,1] were used. For example, $\beta_j(t) = 0.1B_{28,4}$. For non-sparse discriminant function, $\beta_j(t)$ was defined as $\sum_{i=1}^{5}(c_i/i)\phi_i(t)$ where $\phi_i(t) = z_1\sin(z_2\pi it), t \in [0, 1]$. Here, $c = (c_1, c_2, c_3, c_4, c_5)$ and $z = (z_1, z_2)$ are parameters to set. Each $\beta_j(t)$ is discretized with 100 equispaced points within its domain to obtain $\widetilde{\beta}_j$. Next, discretized multivariate mean function for the first class mean, $\widetilde{\boldsymbol{\mu}}_0$, was obtained at once through discretizing $\sum_{i=1}^{5}(q_i/i)\psi_i(t)$ where $\psi_i(t) = \sqrt{2}\sin(\pi it), t \in [0, 1]$ with 300 equispaced points for simplicity.

Table S5 and Table S6 present the parameters used for each setting. Setting 2 is where all parameters are the same as Setting 1 but with unbalanced observations from each class.

Table S5: Parameters for discriminant functions in the simulation study.

	Covariance	Variable	Discriminant function
Setting 1,2	$\boldsymbol{\gamma}_1$	X_1	$0.1B_{28,4}$
		X_2	$-0.15B_{24,4}$
		X_3	$0.1B_{20,4}$
	$\boldsymbol{\gamma}_2$	X_1	$0.05B_{10,4}$
		X_2	$-0.06B_{14,4}$
		X_3	$0.05B_{28,4}$
	$\boldsymbol{\gamma}_3$	X_1	$0.1B_{15,4}$
		X_2	$-0.15B_{26,4}$
		X_3	$0.1B_{10,4}$
Setting 3	$\boldsymbol{\gamma}_1$	X_1	$c = (0.019, -0.006, -0.027, 0.015, -0.011), z = (2, 3)$
		X_2	$c = (0.005, 0.023, 0.003, -0.015, -0.015), z = (3, 2)$
		X_3	$c = (-0.006, -0.025, -0.023, 0.015, -0.007), z = (2.5, 2)$
	$\boldsymbol{\gamma}_2$	X_1	$c = (-0.003, -0.004, -0.009, 0.021, 0.010), z = (2, 3)$
		X_2	$c = (0.002, -0.009, 0.017, 0.001, 0.008), z = (3, 2)$
		X_3	$c = (0.004, 0.005, 0.005, -0.014, -0.005), z = (2.5, 2)$
	$\boldsymbol{\gamma}_3$	X_1	$c = (-0.006, 0.005, 0.012, -0.001, -0.003), z = (2, 3)$
		X_2	$c = (-0.006, 0.006, -0.007, 0.006, -0.003), z = (3, 2)$
		X_3	$c = (-0.006, -0.001, 0.001, -0.009, 0.015), z = (2.5, 2)$
Setting 4	$\boldsymbol{\gamma}_1$	X_1	$0.1B_{22,4}$ and $0.1B_{10,4}$
		X_2	$0.1B_{5,4} - 0.15B_{25,4}$ and $-0.15B_{20,4}$
		X_3	$0.1B_{20,4}$ and $0.1B_{5,4}$
	$\boldsymbol{\gamma}_2$	X_1	$0.05B_{15,4}$ and $0.05B_{8,4}$
		X_2	$-0.05B_{10,4} + 0.1B_{20,4}$ and $0.1B_{22,4}$
		X_3	$-0.05B_{22,4}$ and $-0.1B_{15,4}$
	$\boldsymbol{\gamma}_3$	X_1	$0.1B_{22,4}$ and $0.1B_{10,4}$
		X_2	$0.1B_{5,4} - 0.15B_{25,4}$ and $-0.15B_{20,4}$
		X_3	$0.1B_{20,4}$ and $0.1B_{5,4}$
Setting 5	$\boldsymbol{\gamma}_1$ & $\boldsymbol{\gamma}_2$	X_1	$0.1B_{12,4}$
		X_2	$-0.15B_{21,4}$
		X_3	$0.1B_{5,4}$

Table S6: Parameters for mean functions in the simulation study

	Covariance	Mean function	τ
Setting 1,2	$\boldsymbol{\gamma}_1$	$q = (-0.14, -0.04, -0.05, -0.19, 0.15)$	0.15
	$\boldsymbol{\gamma}_2$	$q = (0.07, -0.17, 0.02, 0.04, 0.33)$	0.07
	$\boldsymbol{\gamma}_3$	$q = (-0.29, -0.95, 0.09, -0.29, -0.77)$	0.2
Setting 3	$\boldsymbol{\gamma}_1$	$q = (-0.14, -0.04, -0.05, -0.19, 0.15)$	0.15
	$\boldsymbol{\gamma}_2$	$q = (-0.14, -0.04, -0.05, -0.19, 0.15)$	0.1
	$\boldsymbol{\gamma}_3$	$q = (-0.14, -0.04, -0.05, -0.19, 0.15)$	0.15
Setting 4	$\boldsymbol{\gamma}_1$	$q = (0.03, -0.04, 0.21, 0.04, -0.01)$	0.1
	$\boldsymbol{\gamma}_2$	$q = (0.03, -0.04, 0.21, 0.04, -0.01)$	0.1
	$\boldsymbol{\gamma}_3$	$q = (0.03, -0.04, 0.21, 0.04, -0.01)$	0.15
Setting 5	$\boldsymbol{\gamma}_1$ & $\boldsymbol{\gamma}_2$	$q = (-0.06, 0.15, -0.13, 0.02, -0.07)$	0.2

Figures S3 - S5 show some sample functions from settings 3, 4, and 5, respectively.

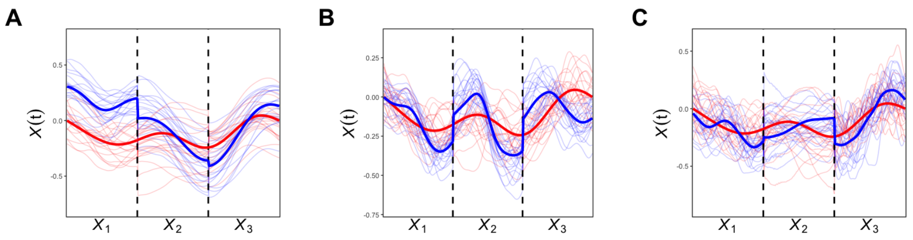

Figure S3: Sample curves from setting 3 with three different covariance structures. **A** Type A. **B** Type B. **C** Type C. Red and blue thick curves are class-wise means.

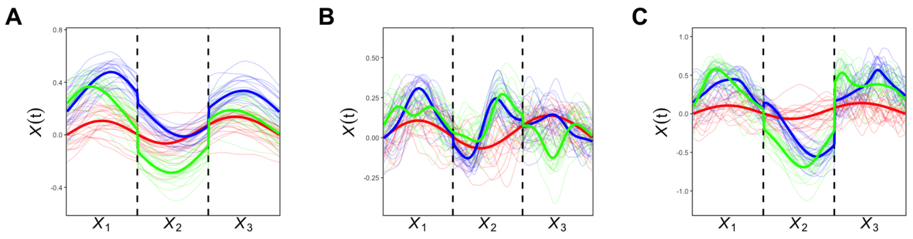

Figure S4: Sample curves from setting 4 with three different covariance structures. **A** Type A. **B** Type B. **C** Type C. Red, blue, and green thick curves are class-wise means.

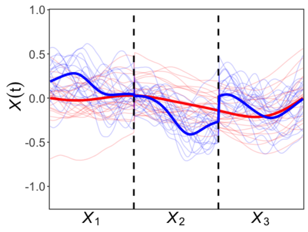

Figure S5: Sample curves from setting 5. Red and blue thick curves are class-wise means.

3 Estimated discriminant function from setting 3

Here, we present the estimated discriminant function from setting 3 where the population discriminant function is non-sparse.

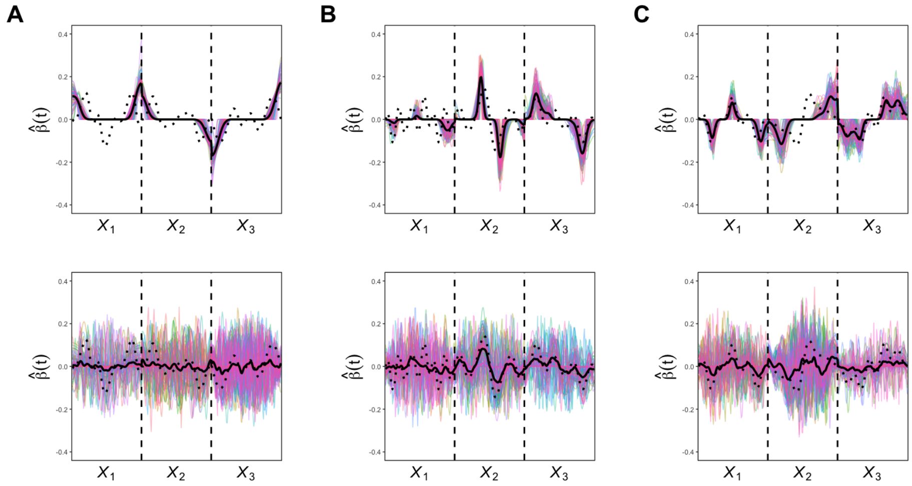

Figure S6: The estimated discriminant functions $\hat{\beta}$ from 100 repetitions for setting 3 with three different covariance structures. **A** Type A. **B** Type B. **C** Type C. Discriminant functions estimated by MV SFLDA are presented in the upper panels and the functions from univariate FLR are presented in the lower panels. Their mean curve is shown in the solid black curve while the true β is in the dotted black curve in each panel.
